# Dysentery

**Published:** 1857-09

**Authors:** 


*Dysentery.*—Dr. J. L. Abernethy, in an article in the Southern Journal of Medical and Physical Sciences, contends that the inflammation of dysentery is not common but specific in its character. The chief reasons adduced for the opinion, is that it has a special locality, viz: the signoid flexure of the color and vicinity; that it prevails epidemically or endemically at certain seasons; that the disease present quite a different lesion from that observed in gastritis, enteritis, &c., and does not yield to treatment ordinarily adapted to inflammation.

